# Supplementary material for: Using the heme peroxidase APEX2 to probe intracellular H2O2 flux and diffusion
Source: Nat Commun. 2024 Feb 9;15:1239. doi: 10.1038/s41467-024-45511-9 (PMC10858230; doi:10.1038/s41467-024-45511-9)
Supplement: Supplementary file 1 — Supplementary Information [file 41467_2024_45511_MOESM1_ESM.pdf]

Supplementary Information

Using the heme peroxidase APEX2 to probe intracellular H_2O_2 flux and diffusion

Mohammad Eid[1,2], Uladzimir Barayeu[1,2], Katerina Sulková[1,2], Carla Aranda-Vallejo[1,2], and Tobias P. Dick[1,2,*]

[1]Division of Redox Regulation, DKFZ-ZMBH Alliance, German Cancer Research Center (DKFZ), Im Neuenheimer Feld 280, 69120 Heidelberg, Germany

[2]Faculty of Biosciences, Heidelberg University, 69120 Heidelberg, Germany

Corresponding author: t.dick@dkfz.de

Supplementary Figures

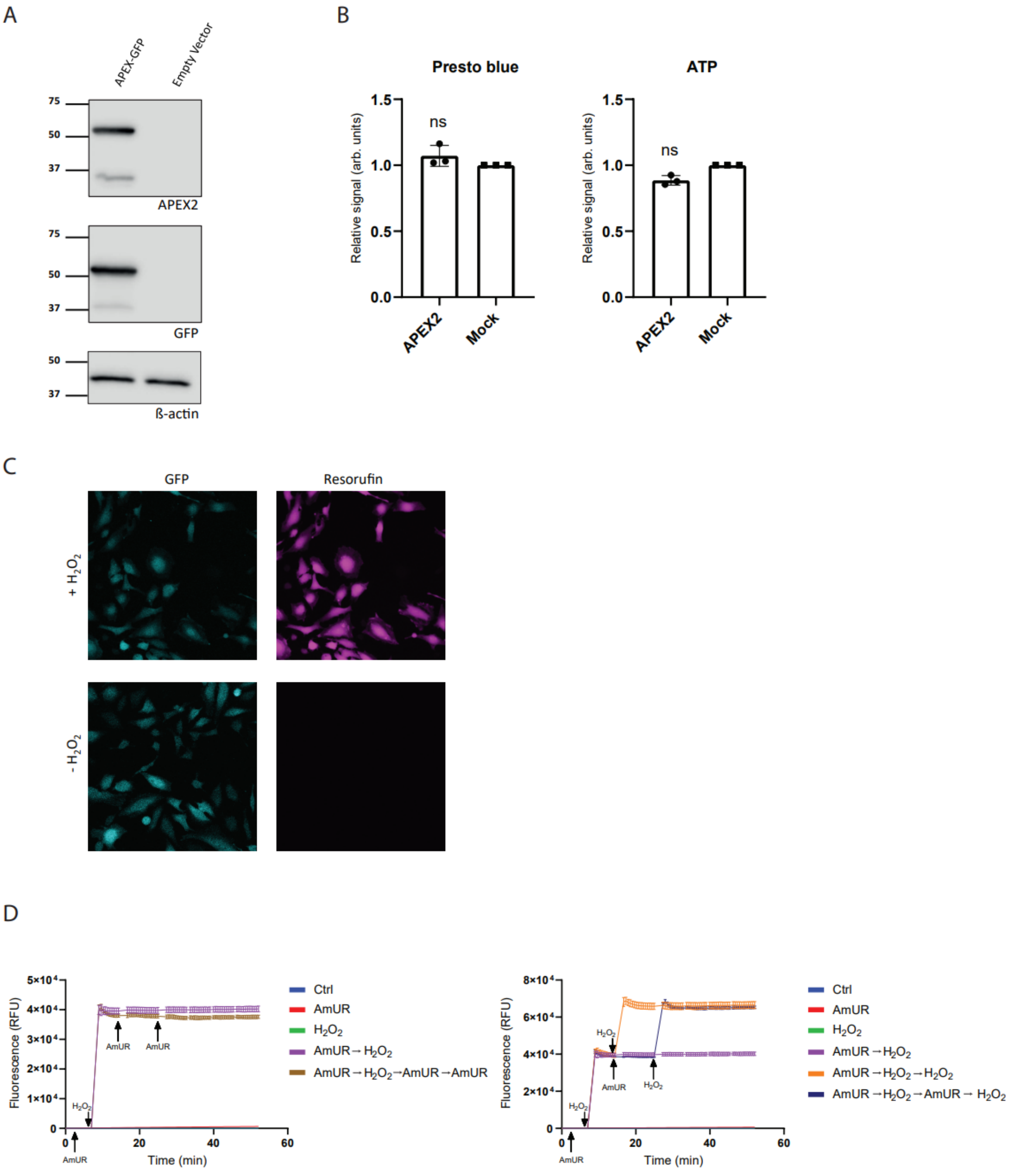

E

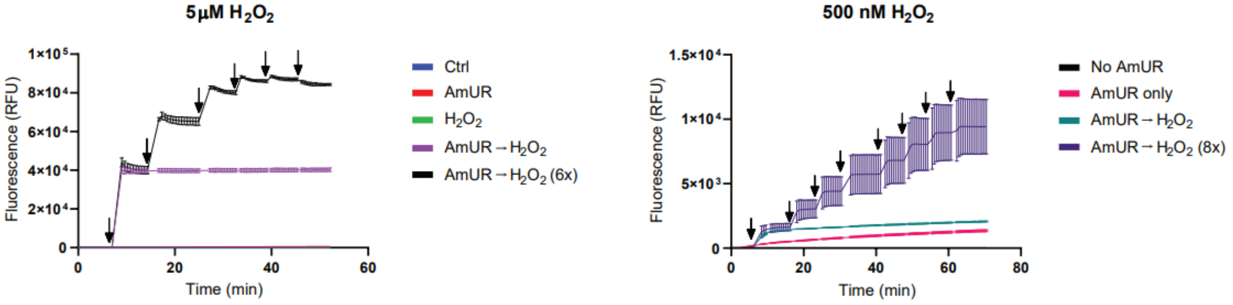
5µM H_2O_2
Fluorescence (RFU)
Time (min)
Ctrl
AmUR
H_2O_2
AmUR→H_2O_2
AmUR→H_2O_2 (6x)
500 nM H_2O_2
No AmUR
AmUR only
AmUR→H_2O_2
AmUR→H_2O_2 (8x)

F

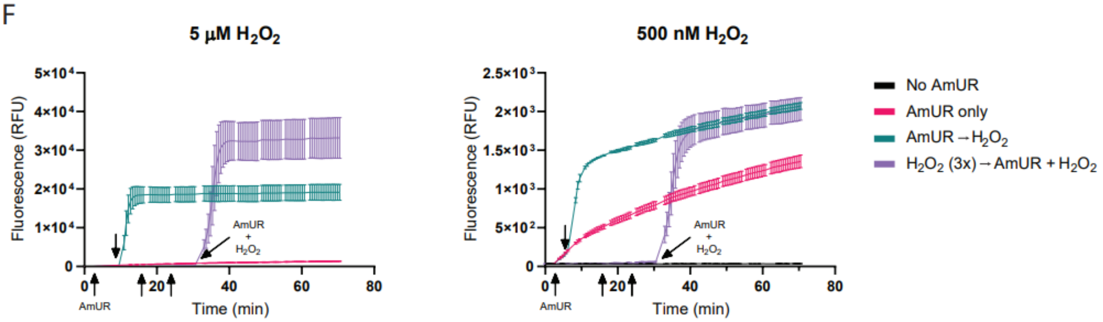
5 µM H_2O_2
500 nM H_2O_2
Fluorescence (RFU)
Time (min)
AmUR
AmUR + H_2O_2
No AmUR
AmUR only
AmUR→H_2O_2
H_2O_2 (3x)→AmUR + H_2O_2

G

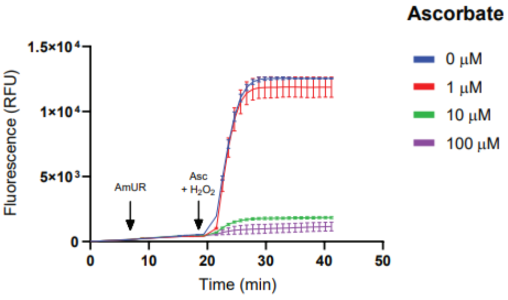
Ascorbate
Fluorescence (RFU)
Time (min)
AmUR
Asc + H_2O_2
0 µM
1 µM
10 µM
100 µM

H

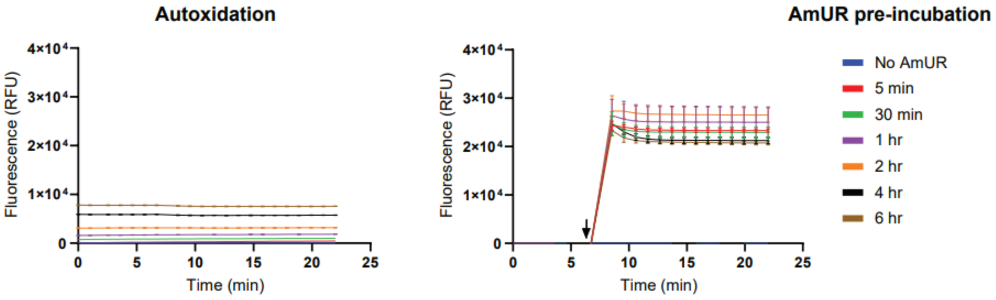
Autoxidation
AmUR pre-incubation
Fluorescence (RFU)
Time (min)
No AmUR
5 min
30 min
1 hr
2 hr
4 hr
6 hr

Supplementary Figure 1. Characterization of the APEX2/AmUR system in HEK293-MSR cells.

(A) Expression of the APEX2-GFP fusion protein in HEK293-MSR cells as demonstrated by immunoblotting with antibodies against APEX2 and GFP. β-actin was used as a loading control. The blot is representative of n=3 independent experiments.

(B) Measurement of reductive capacity (left panel) and ATP levels (right panel) in HEK293-MSR transfected with either APEX2-GFP or empty vector (mock). The results are representative of n=4 independent experiments with n=3 technical replicates each. Error bars represent the mean ± SD. ns: non significant. *P*=0.1964, 0.1000; based on an unpaired t-test.

(C) Fluorescence microscopy of APEX2-GFP-transfected HEK293-MSR cells responding to 10 µM H_2O_2 (upper panels) or solvent control (lower panels). Left panels: GFP fluorescence. Right panels: resorufin fluorescence. The results are representative of n=3 independent experiments.

(D) The fluorescence response is not limited by AmUR availability. Left panel: Following initial treatment of APEX2-expressing HEK293-MSR cells with AmUR (50 µM) and H_2O_2 (5 µM), a second or third addition of AmUR (50 µM) did not lead to any further increase in fluorescence. Right panel: The additional fluorescence increase generated by a second bolus of H_2O_2 (5 µM) is not altered by a second addition of AmUR (50 µM). The results are representative of n=3 independent experiments with n=3 technical replicates each. Error bars represent the mean ± SD.

(E) In principle, the fluorescence response can be limited by AmUR depletion. Following initial treatment of APEX2-expressing HEK293-MSR cells with AmUR (50 µM), the repeated addition of H_2O_2 boli (Left panel: 5 µM; Right panel: 500 nM) eventually leads to 'saturation' of the fluorescence response. The arrows indicate the time points of H_2O_2 addition. The results are representative of n=3 independent experiments with n=3 technical replicates each. Error bars represent the mean ± SD.

(F) APEX2 is not self-inactivating in the presence of H_2O_2 and the absence of AmUR. Three boli of H_2O_2 (Left panel: 5 µM; Right panel: 500 nM) were added to APEX2-expressing HEK293-MSR cells before the addition of AmUR (50 µM). The observed increase in the fluorescence signal implies full APEX2 activity. The unlabeled arrows indicate the time points of H_2O_2 addition (5 µM or 500 nM). The results are representative of n=3 independent experiments with n=3 technical replicates each. Error bars represent the mean ± SD.

(G) The fluorescence response can be inhibited by ascorbate. Following initial treatment of APEX2-expressing HEK293-MSR cells with AmUR (50 µM), ascorbate (0-100 µM) was added, followed by the immediate addition of H_2O_2 (5 µM). The results are representative of n=3 independent experiments with n=3 technical replicates each. Error bars represent the mean ± SD.

(H) Long-term stability of AmUR in cell culture. AmUR (50 µM) was incubated with APEX2-expressing HEK293-MSR cells for the indicated time periods. Left panel: fluorescence caused by autoxidation of AmUR. Right panel: Fluorescence response to the addition of H_2O_2 (5 µM) after subtraction of autoxidation fluorescence. The arrow indicates the time point of H_2O_2 addition. The results are representative of n=3 independent experiments with n=3 technical replicates each. Error bars represent the mean ± SD.

Source data are provided as a Source Data file.

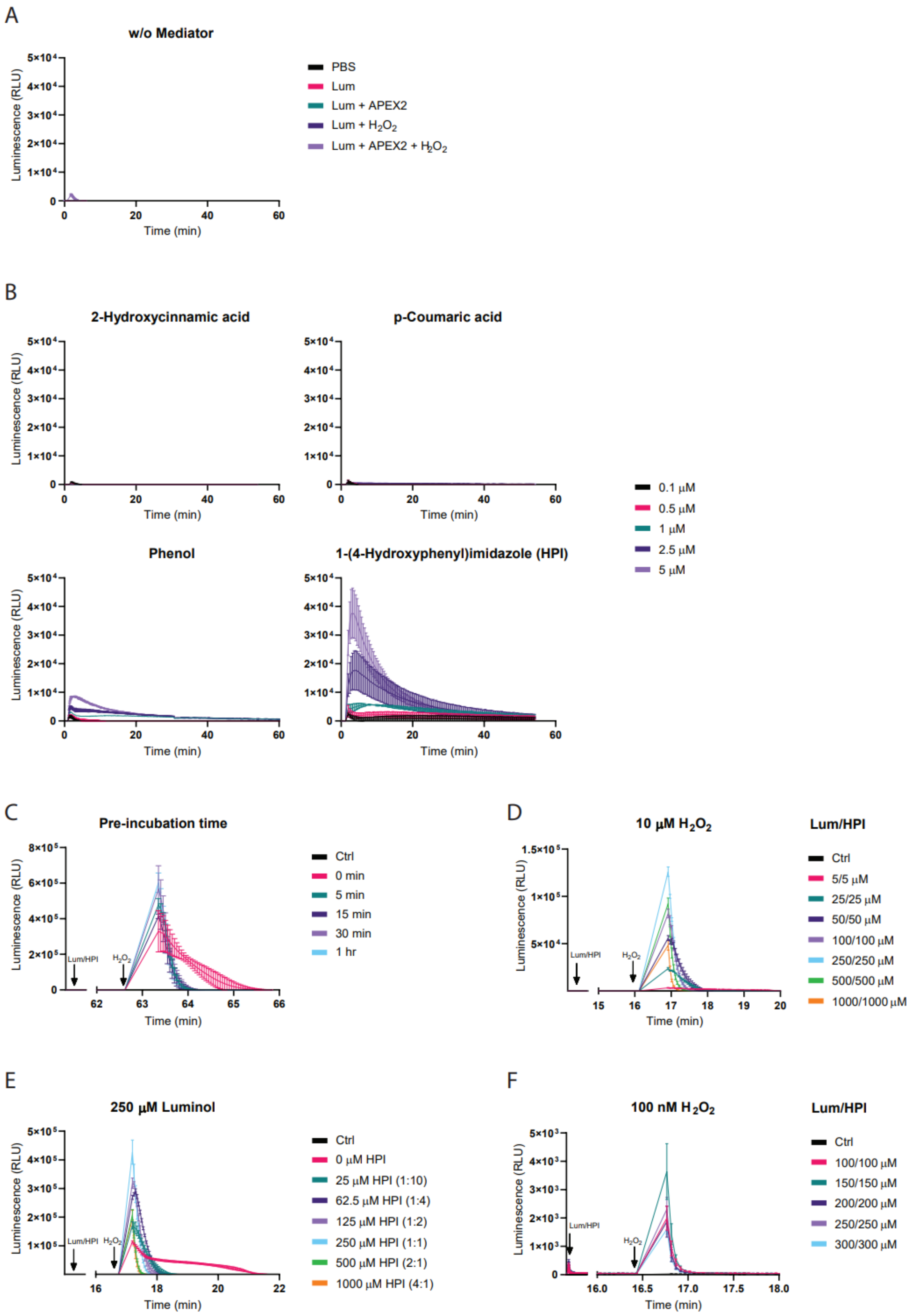

A
w/o Mediator
Luminescence (RLU)
Time (min)
PBS
Lum
Lum + APEX2
Lum + H2O2
Lum + APEX2 + H2O2
B
2-Hydroxycinnamic acid
p-Coumaric acid
Phenol
1-(4-Hydroxyphenyl)imidazole (HPI)
0.1 μM
0.5 μM
1 μM
2.5 μM
5 μM
C
Pre-incubation time
Lum/HPI
H2O2
Ctrl
0 min
5 min
15 min
30 min
1 hr
D
10 μM H2O2
Lum/HPI
Ctrl
5/5 μM
25/25 μM
50/50 μM
100/100 μM
250/250 μM
500/500 μM
1000/1000 μM
E
250 μM Luminol
Ctrl
0 μM HPI
25 μM HPI (1:10)
62.5 μM HPI (1:4)
125 μM HPI (1:2)
250 μM HPI (1:1)
500 μM HPI (2:1)
1000 μM HPI (4:1)
F
100 nM H2O2
Lum/HPI
Ctrl
100/100 μM
150/150 μM
200/200 μM
250/250 μM
300/300 μM

Supplementary Figure 2. Establishing the APEX2/HPI/luminol system.

(A) Luminol is not an efficient substrate for APEX2. Oxidation of luminol (5 μM) by recombinant APEX2 (1.5 nM) in the presence of H_2O_2 (10 μM).

(B) Testing of four phenolic compounds as mediators ('enhancers') of APEX2-dependent luminol oxidation. Oxidation of luminol (5 μM) by recombinant APEX2 (1.5 nM) in the presence of H_2O_2 (10 μM) and the indicated concentration of mediator compound.

(C) Influence of the luminol/HPI pre-incubation time on the cellular luminescence response to 10 μM H_2O_2.

(D) Influence of the luminol/HPI concentration on the cellular luminescence response to 10 μM H_2O_2.

(E) Influence of the luminol/HPI ratio on the cellular luminescence response to 10 μM H_2O_2.

(F) Influence of the luminol/HPI concentration on the cellular luminescence response to 100 nM H_2O_2.

All results in this figure are representative of n=3 independent experiments with n=3 technical replicates each. Error bars represent the mean ± SD. Source data are provided as a Source Data file.

A

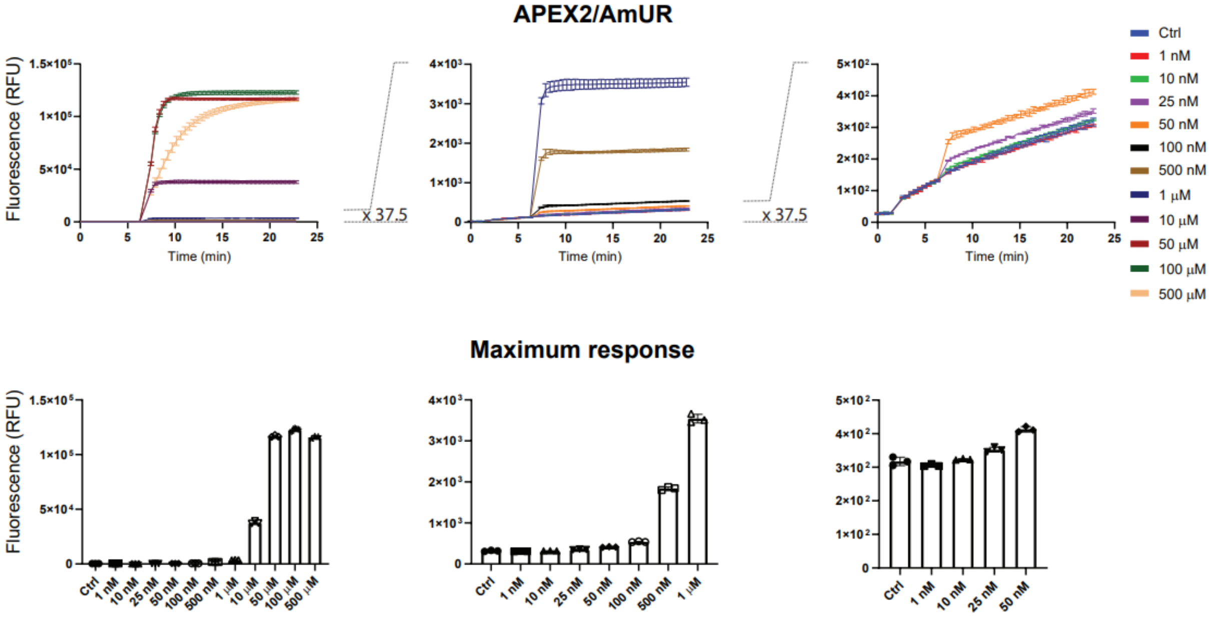
APEX2/AmUR
Fluorescence (RFU)
Time (min)
x 37.5
x 37.5
Ctrl
1 nM
10 nM
25 nM
50 nM
100 nM
500 nM
1 µM
10 µM
50 µM
100 µM
500 µM
Maximum response
Fluorescence (RFU)

B

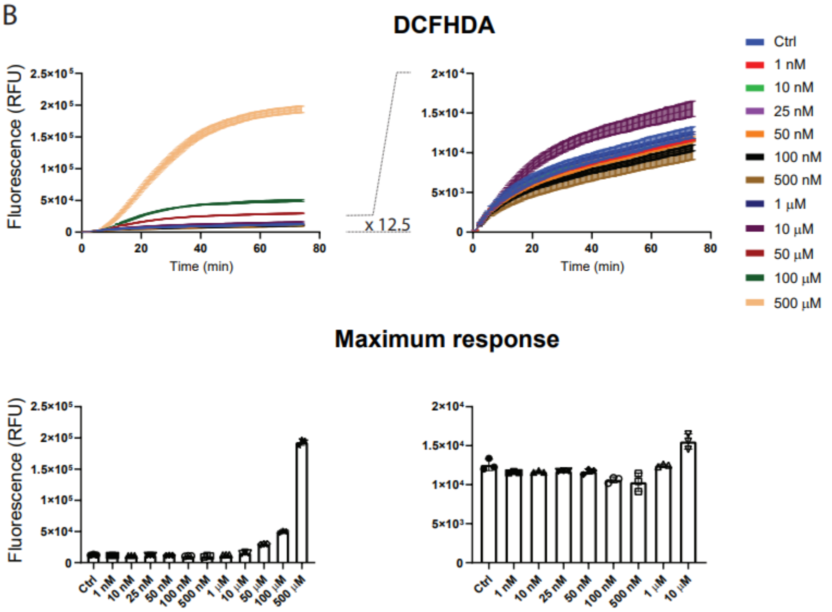
DCFHDA
Fluorescence (RFU)
Time (min)
x 12.5
Ctrl
1 nM
10 nM
25 nM
50 nM
100 nM
500 nM
1 µM
10 µM
50 µM
100 µM
500 µM
Maximum response
Fluorescence (RFU)

C

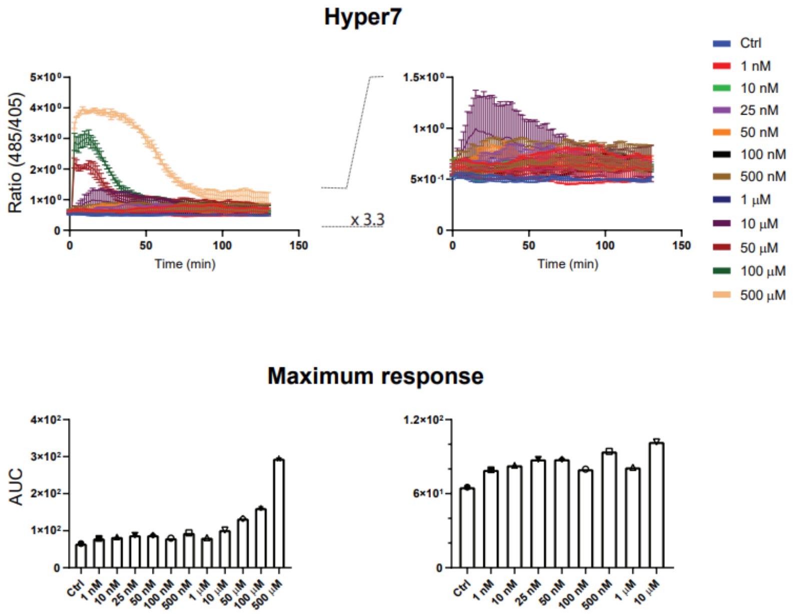

Supplementary Figure 3. A direct comparison of H_2O_2 sensitivity for APEX2, DCFHDA and HyPer7

(A) Upper panels: Fluorescence response of AmUR (50 μM) treated APEX2-expressing HEK293-MSR cells to externally applied H_2O_2 boli (1 nM to 500 μM). Lower panels: corresponding fluorescence maxima.

(B) Upper panels: Fluorescence response of DCFHDA (50 μM) loaded HEK293-MSR cells to externally applied H_2O_2 boli (1 nM to 500 μM). Lower panels: corresponding fluorescence maxima.

(C) Upper panels: Fluorescence response of HyPer7-expressing HEK293-MSR cells to externally applied H_2O_2 boli (1 nM to 500 μM). Lower panels: corresponding fluorescence maxima.

All results in this figure are representative of n=3 independent experiments with n=3 technical replicates each. Error bars represent the mean ± SD. Source data are provided as a Source Data file.

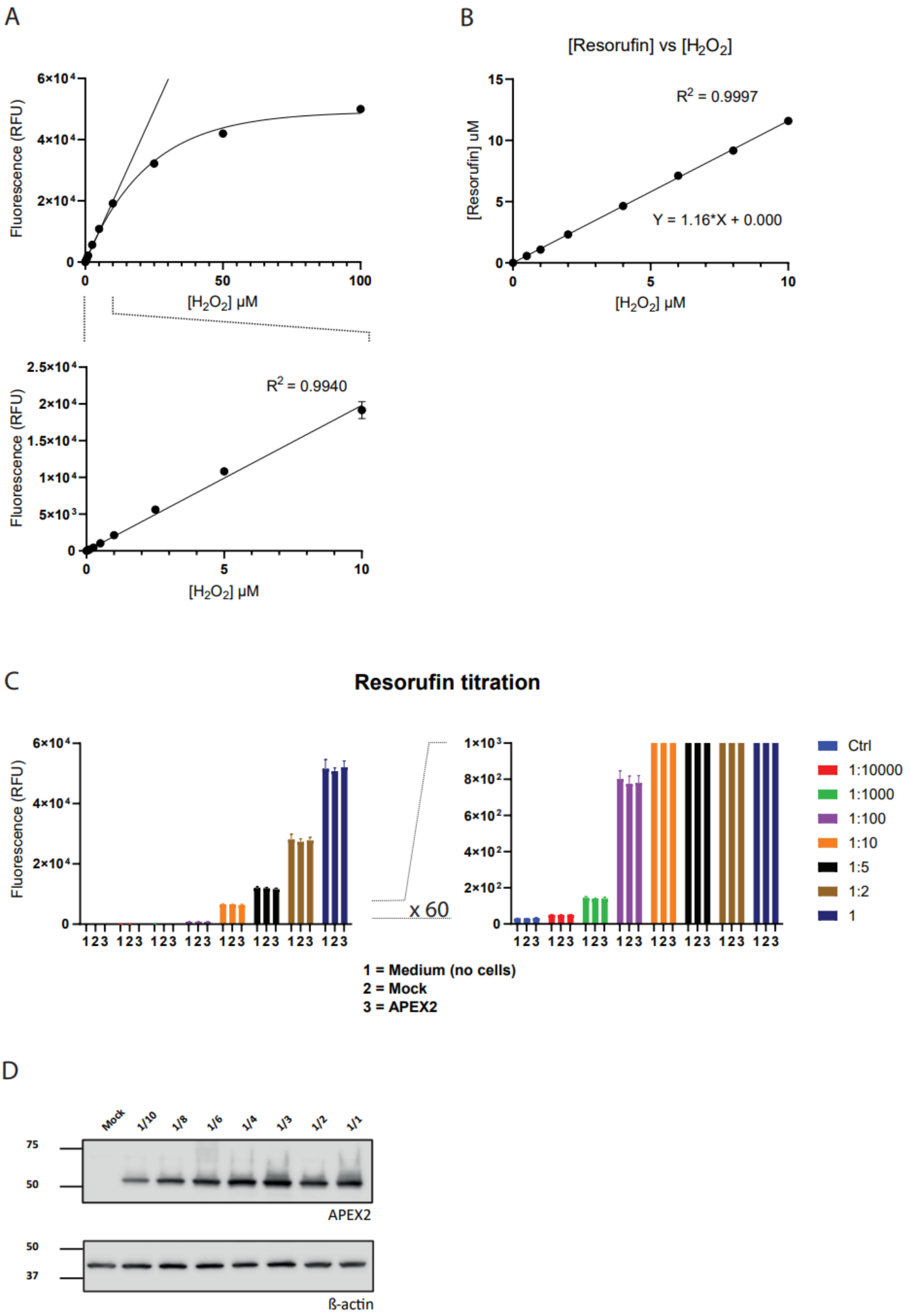
A
Fluorescence (RFU)
6×10⁴
4×10⁴
2×10⁴
0
0
50
100
[H2O2] μM
2.5×10⁴
2×10⁴
1.5×10⁴
1×10⁴
5×10³
0
5
10
R2 = 0.9940
B
[Resorufin] vs [H2O2]
[Resorufin] uM
15
10
5
R2 = 0.9997
Y = 1.16*X + 0.000
C
Resorufin titration
x 60
1×10³
8×10²
6×10²
4×10²
2×10²
1 2 3
Ctrl
1:10000
1:1000
1:100
1:10
1:5
1:2
1
1 = Medium (no cells)
2 = Mock
3 = APEX2
D
Mock
1/10
1/8
1/6
1/4
1/3
1/2
1/1
75
50
APEX2
50
37
ß-actin

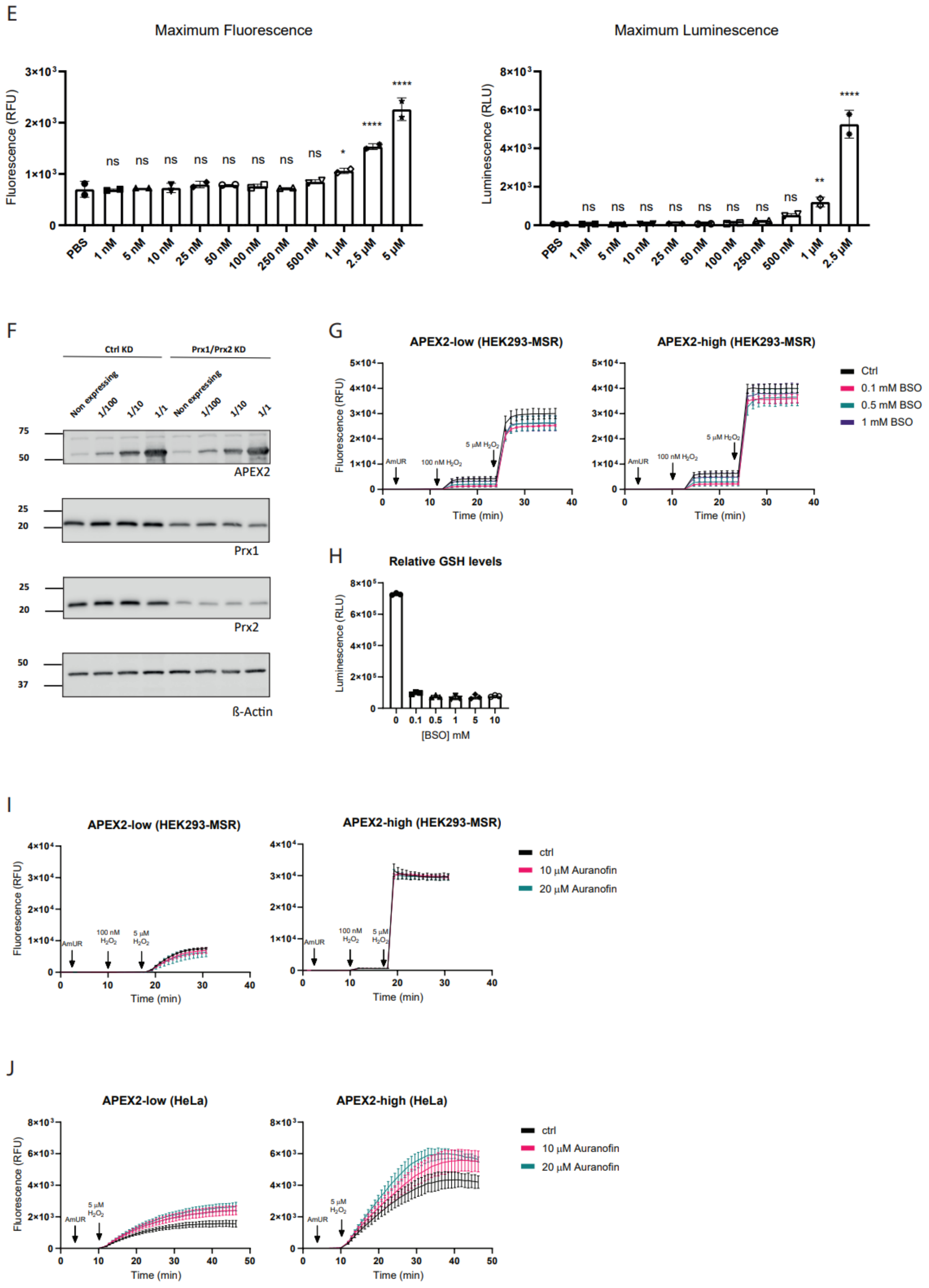

E
Maximum Fluorescence
Fluorescence (RFU)
3×10³
2×10³
1×10³
0
ns
*
****
PBS
1 nM
5 nM
10 nM
25 nM
50 nM
100 nM
250 nM
500 nM
1 µM
2.5 µM
5 µM
Maximum Luminescence
Luminescence (RLU)
8×10³
6×10³
4×10³
2×10³
**
F
Ctrl KD
Prx1/Prx2 KD
Non expressing
1/100
1/10
1/1
75
50
APEX2
25
20
Prx1
Prx2
37
ß-Actin
G
APEX2-low (HEK293-MSR)
APEX2-high (HEK293-MSR)
Fluorescence (RFU)
5×10⁴
4×10⁴
3×10⁴
2×10⁴
1×10⁴
AmUR
100 nM H₂O₂
5 µM H₂O₂
Time (min)
Ctrl
0.1 mM BSO
0.5 mM BSO
1 mM BSO
H
Relative GSH levels
Luminescence (RLU)
8×10⁵
6×10⁵
4×10⁵
2×10⁵
[BSO] mM
I
APEX2-low (HEK293-MSR)
APEX2-high (HEK293-MSR)
Fluorescence (RFU)
Time (min)
ctrl
10 µM Auranofin
20 µM Auranofin
J
APEX2-low (HeLa)
APEX2-high (HeLa)
Fluorescence (RFU)
AmUR
5 µM H₂O₂
Time (min)
ctrl
10 µM Auranofin
20 µM Auranofin

Supplementary Figure 4. Competition between APEX2 and endogenous H_2O_2 consumers

(A) In vitro fluorescence H_2O_2 standard curve using recombinant APEX2 (1.5 µM) and AmUR (50 µM) (upper panel). The relationship is linear up to 10 µM H_2O_2 (lower panel). The results are representative of n=3 independent experiments.

(B) Stoichiometric relationship between H_2O_2 consumption and resorufin formation. The results are representative of n=3 independent experiments.

(C) The presence of cells does not interfere with resorufin fluorescence measurements. Titration of resorufin into medium without cells ('1'), medium with mock-transfected HEK293-MSR cells ('2') and medium with APEX2-expressing HEK293-MSR cells ('3'). The results are representative of n=3 independent experiments with n=3 technical replicates each. Error bars represent the mean ± SD.

(D) Transfection of HEK293-MSR cells with different amounts of APEX2-expressing plasmid. β-actin was used as a loading control. 1/1 corresponds to 2.5 µg of transfected plasmid. The blot is representative of n=3 independent experiments.

(E) Maximum fluorescence (left panel) and luminescence (right panel) obtained upon H_2O_2 titration with 'APEX2-low' HEK293-MSR cells. The detection limit is ≈1 µM H_2O_2. The results are representative of n=4 independent experiments with n=2 technical replicates each. Error bars represent the mean ± SD. *p<0.05; **p<0.01; ****p<0.0001; ns: non significant; Left panel: *P*=0.9998, 0.9996, 0.9996, 0.9112, 0.9366, 0.9925, 0.9996, 0.5530, 0.0123, <0.0001 and <0.0001. Right panel: *P*=>0.9999, >0.9999, >0.9999, 0.9999, >0.9999, 0.9997, 0.9721, 0.3199, 0.0031 and <0.0001; based on a one-way ANOVA.

(F) Partial depletion of Prx1 and Prx2 in HEK293-MSR cells expressing different levels of APEX2. The blot is representative of n=3 independent experiments.

(G) Influence of the γ-glutamylcysteine synthetase inhibitor BSO on APEX2-dependent H_2O_2 turnover in HEK293-MSR cells. Left panel: 'APEX2-low' cells. Right panel: 'APEX2-high' cells. The results are representative of n=3 independent experiments with n=3 technical replicates each. Error bars represent the mean ± SD.

(H) Relative GSH levels in HEK293-MSR cells treated with different concentrations of BSO for 24 hrs. The results are representative of n=3 independent experiments with n=3 technical replicates each. Error bars represent the mean ± SD.

(I) Influence of the TrxR inhibitor auranofin on APEX2-dependent H_2O_2 turnover in HEK293-MSR cells. Left panel: 'APEX2-low' cells. Right panel: 'APEX2-high' cells. The results are representative of n=3 independent experiments with n=3 technical replicates each. Error bars represent the mean ± SD.

(J) Influence of the TrxR inhibitor auranofin on APEX2-dependent H_2O_2 turnover in transiently-transfected HeLa cells. The results are representative of n=3 independent experiments with n=3 technical replicates each. Error bars represent the mean ± SD.

Source data are provided as a Source Data file.

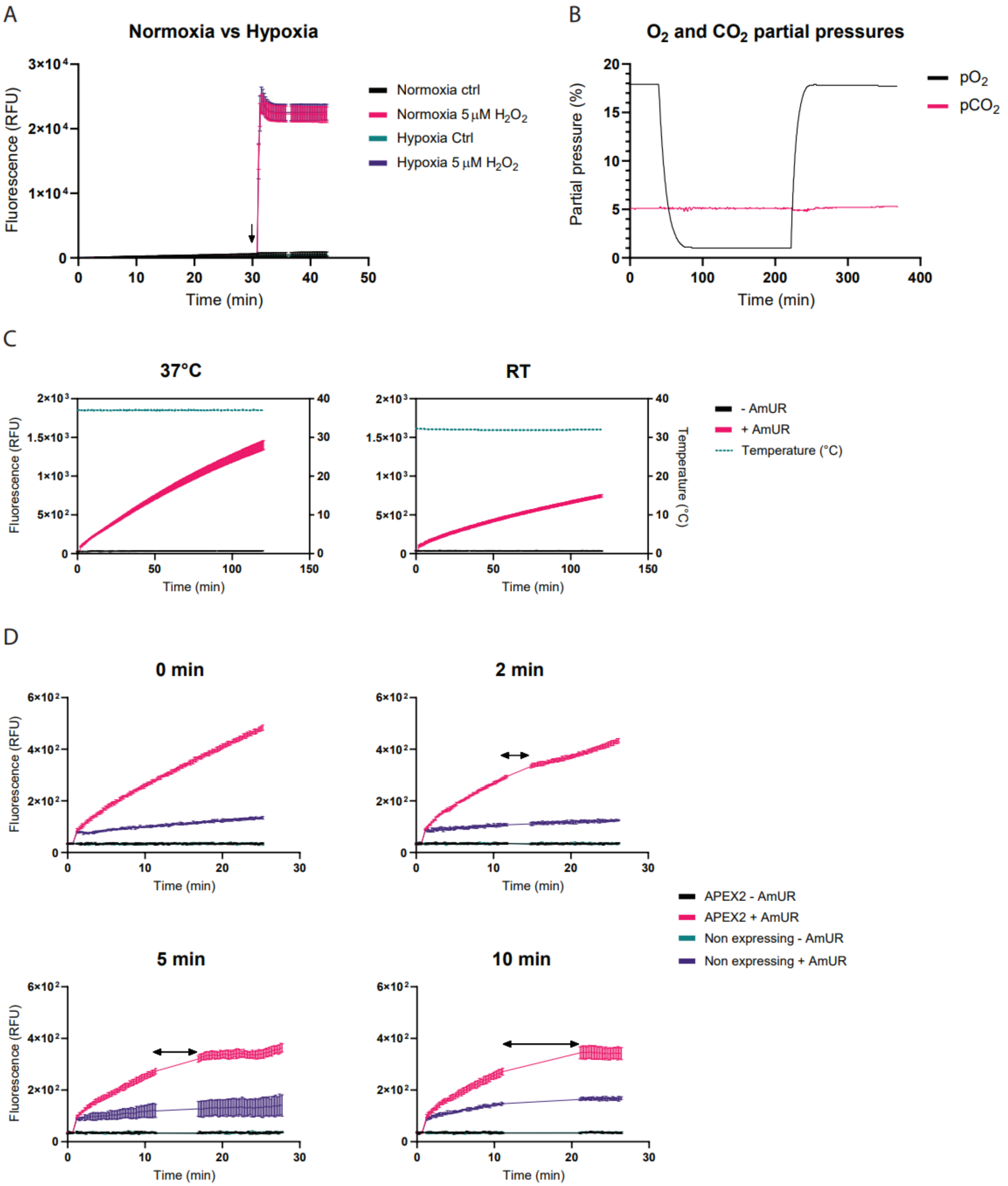

A
Normoxia vs Hypoxia
Fluorescence (RFU)
Time (min)
Normoxia ctrl
Normoxia 5 μM H_2O_2
Hypoxia Ctrl
Hypoxia 5 μM H_2O_2
B
O_2 and CO_2 partial pressures
Partial pressure (%)
Time (min)
pO_2
pCO_2
C
37°C
RT
Fluorescence (RFU)
Temperature (°C)
Time (min)
- AmUR
+ AmUR
Temperature (°C)
D
0 min
2 min
5 min
10 min
Fluorescence (RFU)
Time (min)
APEX2 - AmUR
APEX2 + AmUR
Non expressing - AmUR
Non expressing + AmUR

Supplementary Figure 5.

(A) Influence of hypoxia (1% O_2) vs. normoxia on the fluorescence response in APEX2-expressing HEK293-MSR cells in response to external addition of 5 µM H_2O_2 using 50 µM AmUR. The arrow indicates the time point of H_2O_2 addition. The results are representative of n=3 independent experiments with n=3 technical replicates each. Error bars represent the mean ± SD.

(B) Time course of O_2 and CO_2 partial pressures recorded during the hypoxia-reoxygenation experiment shown in Fig. 5A.

(C) Influence of cell culture temperature (Left panel: 37°C; Right panel: room temperature, RT) on AmUR oxidation (50 µM) in APEX2-expressing HEK293-MSR cells, as caused by endogenous H_2O_2 generation. The results are representative of n=3 independent experiments with n=3 technical replicates each. Error bars represent the mean ± SD.

(D) Influence of acutely cooling APEX2-expressing HEK293-MSR cells for different periods of time (0, 2, 5 and 10 min). The double-headed arrows visualize the period of placing closed plates into a compartment with -20°C air temperature. The results are representative of n=3 independent experiments with n=3 technical replicates each. Error bars represent the mean ± SD.

Source data are provided as a Source Data file.

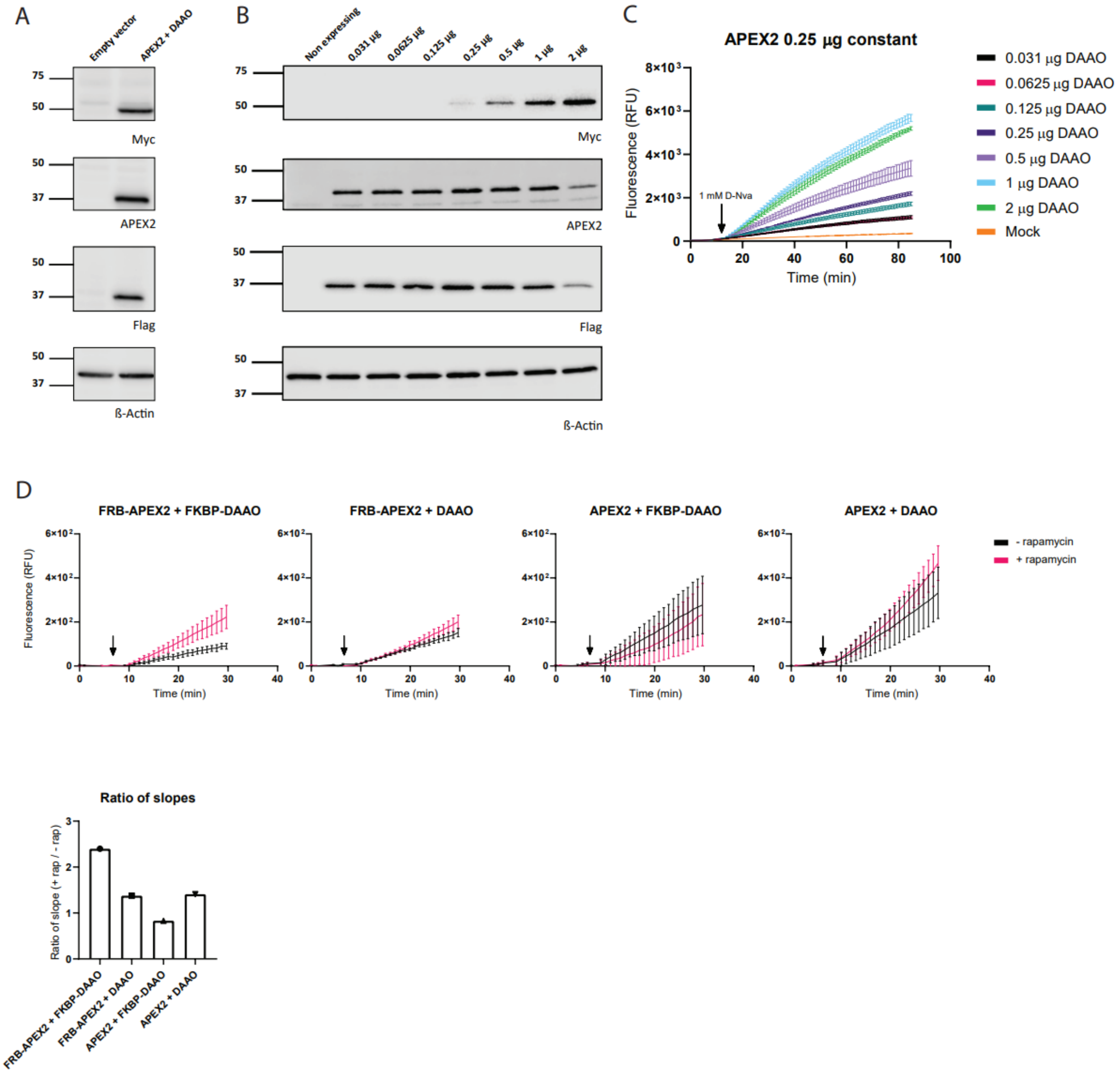

A
Empty vector
APEX2 + DAAO
75
50
Myc
50
37
APEX2
50
37
Flag
50
37
ß-Actin
B
Non expressing
0.031 µg
0.0625 µg
0.125 µg
0.25 µg
0.5 µg
1 µg
2 µg
75
50
Myc
50
37
APEX2
50
37
Flag
50
37
ß-Actin
C
APEX2 0.25 µg constant
Fluorescence (RFU)
8×10³
6×10³
4×10³
2×10³
0
1 mM D-Nva
0
20
40
60
80
100
Time (min)
0.031 µg DAAO
0.0625 µg DAAO
0.125 µg DAAO
0.25 µg DAAO
0.5 µg DAAO
1 µg DAAO
2 µg DAAO
Mock
D
FRB-APEX2 + FKBP-DAAO
FRB-APEX2 + DAAO
APEX2 + FKBP-DAAO
APEX2 + DAAO
Fluorescence (RFU)
6×10²
4×10²
2×10²
0
0
10
20
30
40
Time (min)
- rapamycin
+ rapamycin
Ratio of slopes
Ratio of slope (+ rap / - rap)
3
2
1
0
FRB-APEX2 + FKBP-DAAO
FRB-APEX2 + DAAO
APEX2 + FKBP-DAAO
APEX2 + DAAO

Supplementary Figure 6.

(A) Co-expression of FRB-flag-APEX2 and FKBP-myc-DAAO in HeLa cells, as demonstrated by immunoblotting. The blot is representative of n=3 independent experiments.

(B) Expression of FRB-flag-APEX2 at a given level (0.25 µg transfected plasmid) in combination with expression of FKBP-myc-DAAO at variable levels in HeLa cells, as demonstrated by immunoblotting. The blot is representative of n=3 independent experiments.

(C) Fluorescence response to D-norvalin (D-Nva; 1 mM) in HeLa cells expressing FRB-flag-APEX2 at a fixed level (0.25 µg transfected plasmid) and FKBP-myc-DAAO at various different levels. The results are representative of n=3 independent experiments with n=3 technical replicates each. Error bars represent the mean ± SD.

(D) Upper panels: Fluorescence response of HeLa cells expressing different combinations of DAAO and APEX2 constructs, with and without fused FKBP or FRB, in the presence or absence of rapamycin (100 nM). The arrow indicates the addition of D-Nva (4 mM). Bottom panel: The +/- rapamycin slope ratio obtained for the different combinations.

Source data are provided as a Source Data file.

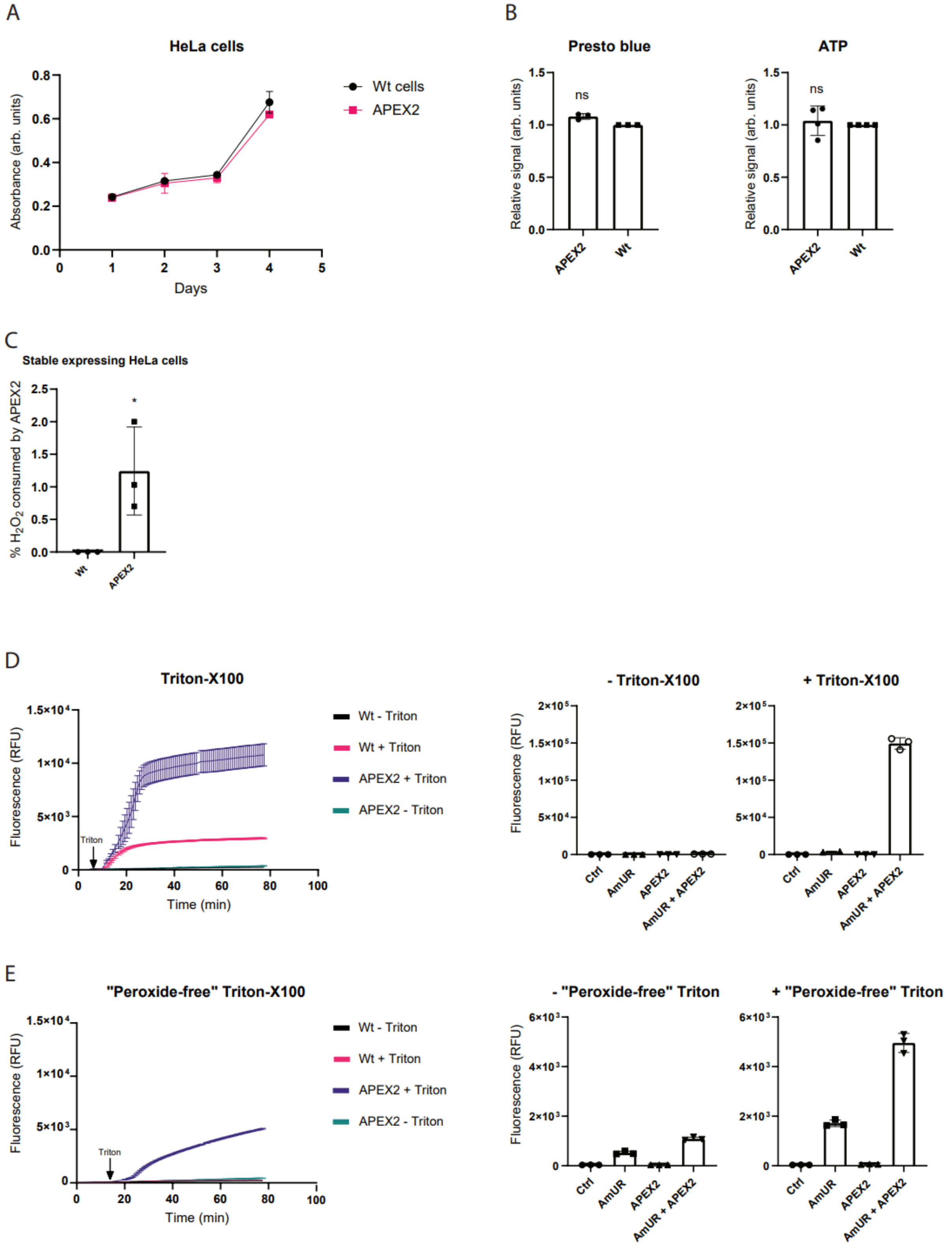
A
HeLa cells
Absorbance (arb. units)
Days
Wt cells
APEX2
B
Presto blue
ATP
Relative signal (arb. units)
ns
APEX2
Wt
C
Stable expressing HeLa cells
% H_2O_2 consumed by APEX2
*
Wt
APEX2
D
Triton-X100
Fluorescence (RFU)
Time (min)
Triton
Wt - Triton
Wt + Triton
APEX2 + Triton
APEX2 - Triton
- Triton-X100
+ Triton-X100
Ctrl
AmUR
APEX2
AmUR + APEX2
E
"Peroxide-free" Triton-X100
- "Peroxide-free" Triton
+ "Peroxide-free" Triton

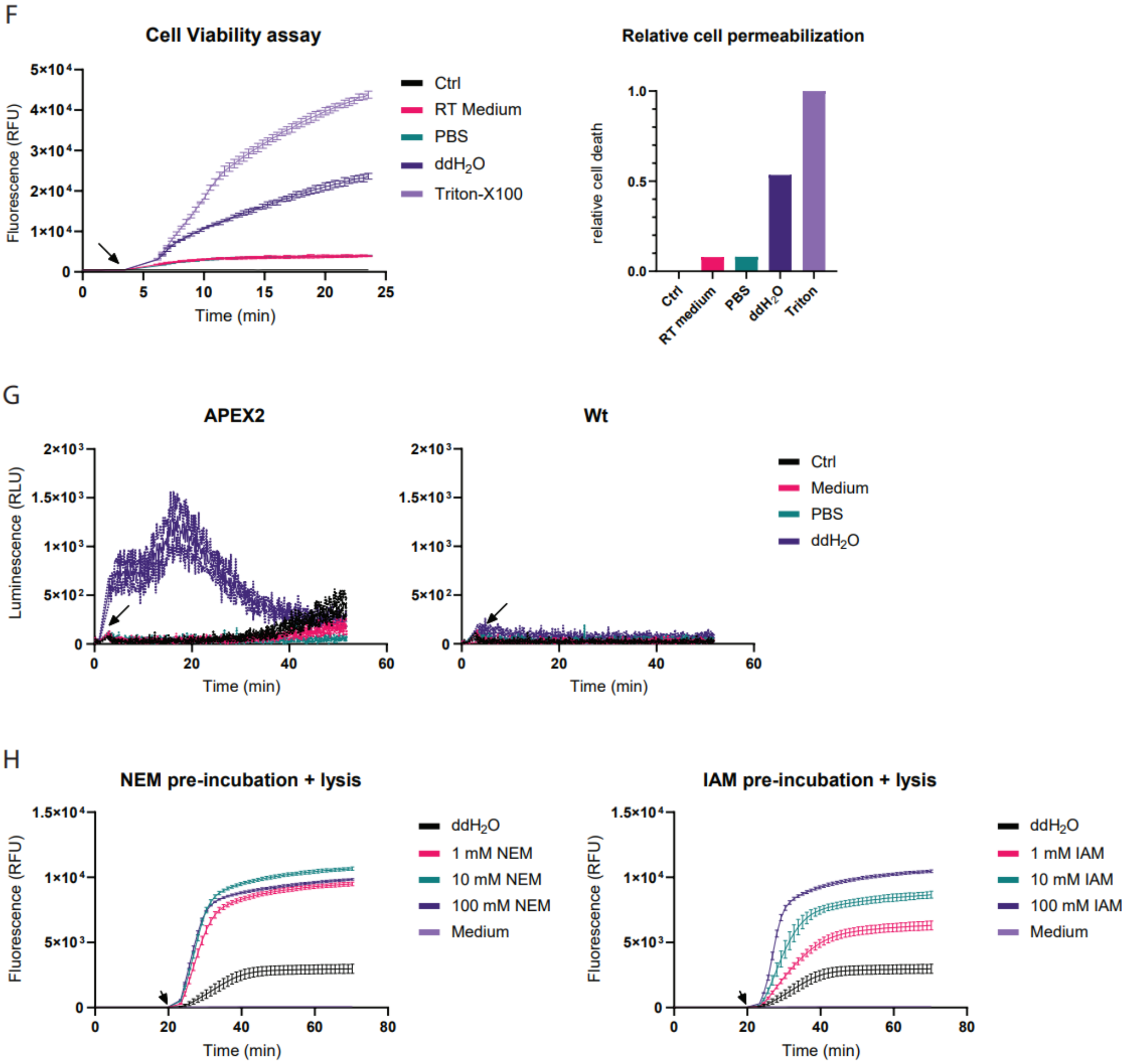
F
Cell Viability assay
Fluorescence (RFU)
5×10⁴
4×10⁴
3×10⁴
2×10⁴
1×10⁴
0
0
5
10
15
20
25
Time (min)
Ctrl
RT Medium
PBS
ddH2O
Triton-X100
Relative cell permeabilization
relative cell death
1.0
0.5
0.0
Ctrl
RT medium
PBS
ddH2O
Triton
G
APEX2
Luminescence (RLU)
2×10³
1.5×10³
1×10³
5×10²
0
0
20
40
60
Time (min)
Wt
2×10³
1.5×10³
1×10³
5×10²
0
0
20
40
60
Time (min)
Ctrl
Medium
PBS
ddH2O
H
NEM pre-incubation + lysis
Fluorescence (RFU)
1.5×10⁴
1×10⁴
5×10³
0
0
20
40
60
80
Time (min)
ddH2O
1 mM NEM
10 mM NEM
100 mM NEM
Medium
IAM pre-incubation + lysis
Fluorescence (RFU)
1.5×10⁴
1×10⁴
5×10³
0
0
20
40
60
80
Time (min)
ddH2O
1 mM IAM
10 mM IAM
100 mM IAM
Medium

Supplementary Figure 7.

(A) Proliferation of HeLa cells with or without stable expression of cytosolic APEX2, based on the Crystal Violet assay. The results are representative of n=3 independent experiments with n=3 technical replicates each. Error bars represent the mean ± SD.

(B) Reductive capacity, based on the PrestoBlue assay (left panel), and ATP levels (right panel) of HeLa cells with or without stable expression of cytosolic APEX2. The results are representative of n=4 independent experiments with n=3 technical replicates each. Error bars represent the mean ± SD. ns: non significant; *P*=0.1000 and 0.5922; based on an unpaired t-test.

(C) Percentage of H_2O_2 consumed by APEX2 in HeLa cells stably expressing cytosolic APEX2. About 1% of exogenously added H_2O_2 is consumed by APEX2 in these cells. Based on n=3 biological replicates. Error bars represent the mean ± SD. *p<0.05; *P*=0.0333, based on an unpaired t-test.

(D) Commercial Triton X-100 preparations are contaminated by peroxides. Left panel: Influence of regular laboratory grade Triton X-100 on HeLa cells stably expressing cytosolic APEX2. Right panels: In vitro response of recombinant APEX2 (1.5 µM) to regular Triton-X100 (1:100). The results are representative of n=3 independent experiments with n=3 technical replicates each. Error bars represent the mean ± SD.

(E) So-called ‘peroxide-free’ Triton X-100 preparations contain lower but still significant amounts of peroxides. Left panel: Influence of ‘peroxide-free’ Triton X-100 on HeLa cells stably expressing cytosolic APEX2. Right panels: In vitro response of recombinant APEX2 (1.5 µM) to ‘peroxide free’ Triton-X100 (1:100). The results are representative of n=3 independent experiments with n=3 technical replicates each. Error bars represent the mean ± SD.

(F) Hypotonic lysis of HeLa cells by double-distilled water (ddH_2O), as monitored by the CellTox Green assay. Triton-X100 (1:100) was used as a positive control. The arrow indicates the time point of medium replacement by ddH_2O. Right panel: Quantification of cell permeabilization in response to the indicated treatments relative to Triton-X100. The results are representative of n=3 independent experiments with n=3 technical replicates each. Error bars represent the mean ± SD.

(G) Luminescence response to hypotonic lysis in HeLa cells expressing (left panel) or not expressing APEX2 (right panel). The arrows indicate the time of medium change. The results are representative of n=3 independent experiments with n=3 technical replicates each. Error bars represent the mean ± SD.

(H) Influence of pre-incubation (for 15 min) with the indicated concentrations of NEM (left panel) or IAM (right panel) on AmUR fluorescence in HeLa cells stably expressing APEX2 following hypotonic lysis. The arrows indicate the time of medium change. The results are representative of n=3 independent experiments with n=3 technical replicates each. Error bars represent the mean ± SD.

Source data are provided as a Source Data file.
