# Supplementary material for: Using the heme peroxidase APEX2 to probe intracellular H2O2 flux and diffusion
Source: Nat Commun. 2024 Feb 9;15:1239. doi: 10.1038/s41467-024-45511-9 (PMC10858230; doi:10.1038/s41467-024-45511-9)
Supplement: Supplementary file 3 — Reporting Summary [file 41467_2024_45511_MOESM3_ESM.pdf]

nature portfolio

Corresponding author(s): Tobias P. Dick

Last updated by author(s): Jan 9, 2024

Reporting Summary

Nature Portfolio wishes to improve the reproducibility of the work that we publish. This form provides structure for consistency and transparency in reporting. For further information on Nature Portfolio policies, see our Editorial Policies and the Editorial Policy Checklist.

Statistics

For all statistical analyses, confirm that the following items are present in the figure legend, table legend, main text, or Methods section.

n/a	Confirmed	
☐	☒	The exact sample size (*n*) for each experimental group/condition, given as a discrete number and unit of measurement
☐	☒	A statement on whether measurements were taken from distinct samples or whether the same sample was measured repeatedly
☐	☒	The statistical test(s) used AND whether they are one- or two-sided<br>*Only common tests should be described solely by name; describe more complex techniques in the Methods section.*
☒	☐	A description of all covariates tested
☒	☐	A description of any assumptions or corrections, such as tests of normality and adjustment for multiple comparisons
☐	☒	A full description of the statistical parameters including central tendency (e.g. means) or other basic estimates (e.g. regression coefficient) AND variation (e.g. standard deviation) or associated estimates of uncertainty (e.g. confidence intervals)
☐	☒	For null hypothesis testing, the test statistic (e.g. *F*, *t*, *r*) with confidence intervals, effect sizes, degrees of freedom and *P* value noted<br>*Give P values as exact values whenever suitable.*
☒	☐	For Bayesian analysis, information on the choice of priors and Markov chain Monte Carlo settings
☒	☐	For hierarchical and complex designs, identification of the appropriate level for tests and full reporting of outcomes
☒	☐	Estimates of effect sizes (e.g. Cohen's *d*, Pearson's *r*), indicating how they were calculated

Our web collection on statistics for biologists contains articles on many of the points above.

Software and code

Policy information about availability of computer code

Data collection	PHERAstar and CLARIOstar (v.4.00 and v.5.20, BMG), Labscribe (v.4, WPI), ZEN (3.2, blue edition), Image studio (v.5.2)
Data analysis	Mars (v.2.40,BMG), Graphpad Prism (v.8), Labscribe (v.4, WPI), ZEN (3.2, blue edition), ImageJ (1.8.0)

For manuscripts utilizing custom algorithms or software that are central to the research but not yet described in published literature, software must be made available to editors and reviewers. We strongly encourage code deposition in a community repository (e.g. GitHub). See the Nature Portfolio guidelines for submitting code & software for further information.

Data

Policy information about availability of data

All manuscripts must include a data availability statement. This statement should provide the following information, where applicable:

- Accession codes, unique identifiers, or web links for publicly available datasets
- A description of any restrictions on data availability
- For clinical datasets or third party data, please ensure that the statement adheres to our policy

All data generated in this study are included in the main text or the supplementary information. Source data are provided with this paper.

Research involving human participants, their data, or biological material

Policy information about studies with human participants or human data. See also policy information about sex, gender (identity/presentation), and sexual orientation and race, ethnicity and racism.

Reporting on sex and gender	N/A
Reporting on race, ethnicity, or other socially relevant groupings	N/A
Population characteristics	N/A
Recruitment	N/A
Ethics oversight	N/A

Note that full information on the approval of the study protocol must also be provided in the manuscript.

Field-specific reporting

Please select the one below that is the best fit for your research. If you are not sure, read the appropriate sections before making your selection.

☒ Life sciences ☐ Behavioural & social sciences ☐ Ecological, evolutionary & environmental sciences

For a reference copy of the document with all sections, see nature.com/documents/nr-reporting-summary-flat.pdf

Life sciences study design

All studies must disclose on these points even when the disclosure is negative.

Sample size	Generally, experiments involving cells were repeated independently on different days using cells of different passage numbers at least 3 times (biological replicates). For each biological replicate, the mean derived from technical replicates (n=3) is shown as an individual data point. For some kinetic measurements, we show one biological replicate in a main/supplementary figure and the data is shown as the mean of technical replicates (n=3), but for these experiments we have at least 3 biological replicates.
Data exclusions	No data were excluded from analysis
Replication	The experiments were replicated independently at least three times.
Randomization	No experimental groups were used in this study. Randomization was therefore not relevant.
Blinding	Group allocation and binding was not relevant in this study.

Reporting for specific materials, systems and methods

We require information from authors about some types of materials, experimental systems and methods used in many studies. Here, indicate whether each material, system or method listed is relevant to your study. If you are not sure if a list item applies to your research, read the appropriate section before selecting a response.

Materials & experimental systems

n/a	Involved in the study	
☐	☒	Antibodies
☐	☒	Eukaryotic cell lines
☒	☐	Palaeontology and archaeology
☒	☐	Animals and other organisms
☒	☐	Clinical data
☒	☐	Dual use research of concern
☒	☐	Plants

Methods

n/a	Involved in the study	
☒	☐	ChIP-seq
☒	☐	Flow cytometry
☒	☐	MRI-based neuroimaging

Antibodies

Antibodies used	Antibodies used were chicken anti-APEX2 (Innovagen, 126-146), mouse anti-ß-Actin (Sigma, A5441), mouse anti-flag tag (Sigma,

Antibodies used	F3165), mouse anti-GFP (Sigma, G1546), rabbit anti-Prx1 (Cell Signaling, #8499), rabbit anti-Prx2 (Abcam, 109367) and rabbit anti-myc tag (Cell Signaling, 2278). All primary antibodies were used at a dilution of 1:1000, except for mouse anti-flag which was used at 1:30000.<br><br>The HRP-conjugated secondary antibodies used for immunoblotting were anti-chicken (Jackson ImmunoResearch, 703-035-155), anti-mouse (Jackson ImmunoResearch, 115-035-146) and anti-rabbit (Jackson ImmunoResearch, 111-035-144), all used at a dilution of 1:10000. The fluorescent secondary antibodies used were IRDye 800CW donkey anti-chicken (Li-Cor, 926-32218), used at 1:5000, and IRDye 680RD goat anti-mouse (Li-Cor, 926-68070), used at 1:10000.
Validation	All antibodies used for western blots are well-established antibodies. Band patterns were consistent with the manufacturer product information as well as published data.

Eukaryotic cell lines

Policy information about cell lines and Sex and Gender in Research

Cell line source(s)	HeLa and Phoenix Ampho cells (ATCC), HEK293-MSR cells (GripTiteTM, Thermo Fisher Scientific)
Authentication	Cell lines were authenticated via SNP-based Multiplex Cell Line Authentication (www.multiplexion.de)
Mycoplasma contamination	Cell cultures were repeatedly tested negative for Mycoplasma contamination by PCR.
Commonly misidentified lines (See ICLAC register)	No commonly misidentified cell lines were used.

Plants

Seed stocks	N/A
Novel plant genotypes	N/A
Authentication	N/A
